# Feeding regimens reshape rumen microbiota and metabolome in Shorthorn cattle: a multi-omic insight into microbial diversity and metabolic pathway dynamics

**DOI:** 10.3389/fmicb.2025.1657402

**Published:** 2025-10-29

**Authors:** SiFan Dai, ShiChun He, ShuSheng Zhao, Qing Li, HuaMing Mao, DongWang Wu

**Affiliations:** College of Animal Science and Technology, Yunnan Agricultural University, Kunming, China

**Keywords:** Shorthorn cattle, rumen microbial community, metabolomics, different feeding methods, 16S rRNA/ITS, multi-omics

## Abstract

The rumen microbiome plays a central role in ruminant nutrition and health. To investigate the effects of different feeding regimens on it, this study employed multi-omics analysis to reveal how natural grazing versus intensive feeding alters the rumen microbiota and metabolites in Shorthorn cattle. A total of 18 male shorthorn cattle of about 17 months of age and similar body weight were selected and randomly divided into 3 groups: natural grazing bull group (DJCF), intensive feeding bull group (DJCY) and intensive feeding steer group (DJC). The experiment period was 361 days. After the fattening trial, rumen fluid was collected at slaughter. Microbiota and metabolites were analyzed by 16S rRNA sequencing and LC–MS, and correlations were assessed. The results indicate that different feeding regimens were strongly associated with shifts in rumen microbial diversity and community composition. The ACE and Shannon indices of DJCF group were significantly higher than those of DJCY and DJC group (*p* < 0.05). Bacteroidetes and Firmicutes were the dominant phyla, with relative abundances of 57.62% (DJCF), 54.11% (DJCY), 48.84% (DJC) and 34.07, 38.31, 43.08%, respectively, showing no significant differences (*p* > 0.05). At the genus level, *Prevotella* and *Rikenellaceae_RC9_gut_group* were dominant. The abundance of *Prevotella* was highest in DJCY (22.52%), significantly differing from DJC (12.43%; *p* < 0.05), while *Rikenellaceae_RC9_gut_group* abundances were 12.56% (DJCF), 9.92% (DJCY), and 11.89% (DJC). In the fungal community, Neocallimastigomycota and Ascomycota were the dominant phyla, and there were no significant differences among the three groups. At the genus level, *Caecomyces*, the highest in the DJC group, with a significant difference from the DJCF group (*p* < 0.05). *Orpinomyces*, the highest in the DJCF group, with significant differences from the DJCY and DJC groups (*p* < 0.05). There were significant differences in rumen metabolites between different groups, and a variety of different metabolites were identified, involving sucrose and starch metabolism, purine metabolism and other pathways (*p* < 0.05). In addition, there was a significant correlation between rumen microbes and metabolites (*p* < 0.05). Thus, an intensive feeding system altered the rumen microbiome, resulting in improvements of Shorthorn cattle growth. Nevertheless, the specific causal relationships and underlying regulatory mechanisms governing the interplay between rumen microbiota and metabolic processes remain to be further elucidated through in-depth investigations.

## 1 Introduction

The Shorthorn cattle, a prominent breed of beef cattle, originated in the historic counties of Northumberland, Durham, Yorkshire, and Lincolnshire in northeastern England. Empirical evidence suggests that through systematic selective breeding during the late Victorian agricultural revolution, Shorthorn cattle evolved into a globally recognized beef genotype by the early 20th century—a period characterized by proto-industrialization (Deerry, 2018). In comparison to indigenous Chinese cattle breeds, Shorthorn cattle demonstrate superior growth rates and meat quality. This makes them an essential genetic resource for enhancing beef production in China, particularly through hybrid breeding programs.

Traditionally, these cattle are raised on artificial grasslands. However, during the six-month forage withering period, their weight experiences a significant decline, which adversely affects subsequent fattening efficiency. Therefore, it is essential to optimize feeding strategies to mitigate these seasonal nutritional deficits in order to sustain Shorthorn cattle production. By aligning feed composition with the physiological needs of the cattle, intensive feeding regimes can reduce nutrient wastage, enhance metabolic efficiency, and minimize risks of environmental contamination (Moorby and Fraser, 2021).

The microbial ecosystem in the rumen plays a vital role in nutrient digestion and metabolism, with its composition influenced by factors such as host genetic background, developmental stage, dietary composition, and environmental conditions (Lin et al., 2019; Zhang et al., 2018). Changes in dietary nutrient composition can alter the abundance of specific microbial populations. For instance, studies have shown that the energy level of the ration significantly affects the rumen microbial composition in yaks (Ahmad et al., 2020). Additionally, feedlot fattening has been found to impact rumen fermentation, microbial diversity, and meat quality (Hu et al., 2021). Furthermore, Yang et al. (2024) observed that intensive feeding regimes led to an increase in metabolites associated with amino acid metabolism, such as L-glutamate (Yang et al., 2024). The proportion of dietary concentrate has also been reported to influence rumen microbial abundance, diversity, metabolite concentrations, and metabolic pathways (Pang et al., 2022). To gain deeper insights into these diet-driven microbial and metabolic dynamics, multi-omics approaches, integrating genomic, transcriptomic, and metabolomic data, have proven essential. These methods help link functional gene activity with microbial communities and metabolic processes within the rumen (Denman et al., 2018). By applying these approaches, researchers can better understand how dietary alterations affect microbial populations and metabolic functions, leading to a more comprehensive understanding of the rumen ecosystem in ruminants.

Despite significant advancements in ruminant nutrition, Shorthorn cattle, with their distinct genetic and metabolic characteristics, remain underexplored. Most existing studies on Shorthorn cattle have focused on genetic traits and growth performance, often overlooking the functional role of rumen microbiota and metabolites in nutrient utilization. To address this gap, our study integrates 16S/ITS sequencing and LC–MS metabolomics to investigate: (1) the impact of natural grazing versus intensive feeding on rumen microbial diversity and metabolite profiles in Shorthorn cattle, and (2) the relationships between microbial communities and key metabolites and metabolic pathways. This research represents the first multi-omics characterization of the Shorthorn cattle rumen ecosystem, providing valuable insights that can help refine feeding strategies and improve production efficiency.

## 2 Materials and methods

### 2.1 Animals, diets, and experimental design

The study was conducted at the Yunnan Provincial Breeding and Promotion Centre for Breeding Sheep in Xundian County, located in the eastern suburbs of Kunming, approximately 90 km from the city (longitude 103°11′ east, latitude 25°40′ north). The site is situated in a low hilly area at an altitude of 2,040 m, with an average annual temperature of 13.4 °C and average annual precipitation of 1,025 mm. A total of 18 male Shorthorn cattle, approximately 17 months old and with similar body weights, were selected and randomly assigned to three groups: the natural grazing bull group (DJCF, *n* = 6), the intensive feeding bull group (DJCY, *n* = 6), the intensive feeding castrated bull group (DJC, *n* = 6). Castration was performed in early March to allow full recovery before the commencement of the fattening trial. The DJCF group grazed on artificially improved grassland, while the DJCY and DJC groups were raised in enclosures and freely fed a total mixed ration (TMR). All three groups were provided with ad libitum access to water. The nutritional composition of the DJCY group and DJC group are shown in Table 1. The experimental period lasted 361 days, including a 10-day pretest phase and a 351-day main testing phase. During the pre-feeding period, bulls and castrated bulls in the intensive feeding groups were gradually acclimated to the TMR diet. Rumen fluid was collected at slaughter following the completion of the fattening trial.

**Table 1:** Dietary nutrient level in fattening group (Absolute dry base, %).

DM	CP	EE	Ash	Ca	P	NDF	ADF	ADL
13.16	10.79	3.08	3.91	0.7	0.33	63.87	26.28	4.73

### 2.2 Sample collection and measurements

On the day preceding the conclusion of the experiment, all experimental animals were subjected to a 24-h feed withdrawal, followed by an 8-h water restriction, in order to standardize the conditions prior to the collection of rumen fluid. Following the guidelines outlined in the Chinese National Standard GB/T 19477–2004 for bovine slaughtering protocols, qualified personnel performed dissection of the gastrointestinal tract. The rumen was quickly removed to collect the rumen fluid. After filtration through sterile gauze, rumen fluid was aliquoted into three 10 mL cryovials, snap-frozen in liquid nitrogen, and stored at −80 °C for microbiota and metabolite analysis.

### 2.3 DNA extraction and sequencing

DNA was extracted from the microbial community in rumen fluid, stored at −80 °C, using the TIANamp Stool DNA Kit (TianGen, Beijing, China, catalog: DP712), following the manufacturer’s protocol. The rumen fluid samples were subsequently transported under cryogenic conditions to Yunnan Pulis Biotechnology Co., Ltd. (Kunming, China) for high-throughput sequencing. The 16S rRNA and ITS genes from target regions were PCR-amplified with region-specific primers containing unique barcodes, using barcoded specific primers 515F (515F:5’-GTGYCAGCMGCCGCGGTAA-3′) and 806R (5′- GTGCCAGCMGCCGCGG-3′) to amplify the V3 – V4 hypervariable region of 16S rRNA gene. ITS1 region primers (ITS1-5F: GGAAGTAAAAGTCGTAACAAGG, GCTGCGTTCTTCATCGATGC): Identification of fungal diversity. PCR reaction system and conditions: Each PCR reaction system contains 15 μL of Phusion® High-Fidelity PCR Master Mix (New England Biolabs), 0.2 μM primers, and 10 ng of genomic DNA template. The first denaturation step was performed at 98 °C for 1 min, followed by 30 cycles at 98 °C (10 s), 50 °C (30 s), and 72 °C (30 s), with a final extension at 72 °C for 5 min. PCR products were detected by 2% agarose gel electrophoresis. Qualified PCR products were then purified using magnetic beads and quantified via enzyme-linked immunosorbent assay (ELISA). PCR products were mixed in equal volumes according to their concentrations, thoroughly mixed, and subsequently detected by 2% agarose gel electrophoresis. The target band was then recovered using a universal DNA purification and recovery kit (Tian Gen). Library construction was performed using the NEB Next® Ultra™ II FS DNA PCR-free Library Prep Kit (New England Biolabs). The constructed libraries were quantified using Qubit and qPCR. Qualified libraries were sequenced using the NovaSeq 6,000 sequencing platform with PE250 sequencing.

Individual sample data were extracted from the raw sequencing data based on barcode sequences and PCR amplification primers. After demultiplexing, barcode and primer sequences were trimmed to obtain preliminary sequence data. Using FLASH (Version 1.2.11^1^), reads from each sample were merged to generate raw tag data (Raw Tags). Paired-end reads were merged and subjected to quality control with fastp (v0.23.1) to yield high-quality clean tags. Chimeric sequences were identified and removed using UCHIME with reference to the SILVA database for 16S rRNA and the UNITE database for ITS (Knight, 2011). Quality-filtered, non-chimeric sequences were clustered into operational taxonomic units (OTUs) at 97% sequence identity in QIIME 2 (via VSEARCH) (Callahan et al., 2019), and an OTU table and representative sequences were generated (Wang et al., 2021). Taxonomic annotation was conducted in QIIME 2 against the SILVA (v138.1) reference database for 16S rRNA and UNITE (v8.2) for ITS. A phylogenetic tree was inferred from multiple-sequence-aligned OTU representative sequences. Prior to downstream analyses, samples were rarefied to the minimum sequencing depth to standardize library sizes.

### 2.4 Determination and analysis of rumen metabolome

Rumen metabolomics was conducted using LC–MS/MS with a quadrupole-Orbitrap mass spectrometer (Q Exactive Orbitrap) coupled to UHPLC (Thermo Fisher Scientific, USA). Transfer 100 μL of rumen fluid to a 1.5 mL microcentrifuge tube. Add 400 μL of extraction solvent (acetonitrile:methanol, 1:1, v/v) containing an isotopically labeled internal standard mixture. Vortex for 30 s, sonicate in an ice–water bath for 5 min, and incubate at −40 °C for 1 h to precipitate proteins. Centrifuge at 12,000 rpm for 15 min at 4 °C. The resulting supernatant was transferred to a clean glass vial for downstream analyses. A pooled QC sample was prepared by mixing equal aliquots of supernatant from each sample. Polar metabolite profiling was conducted using a Vanquish UHPLC system coupled to an Orbitrap Exploris 120 mass spectrometer (Thermo Fisher Scientific). Chromatographic separation was performed on a Waters ACQUITY UPLC BEH Amide column (2.1 × 50 mm, 1.7 μm) with solvent A (25 mM ammonium acetate + 25 mM ammonium hydroxide in water) and solvent B (acetonitrile). The autosampler was held at 4 °C, and 2 μL were injected. Data acquisition was carried out on an Orbitrap Exploris 120 under Xcalibur v4.4 control (Thermo Fisher Scientific).

Raw data were converted to mzXML with ProteoWizard (msConvert). Metabolite identification used BiotreeDB (v3.0) in R, and results were visualized with R package (Smoot et al., 2011). Differential metabolites were defined by OPLS-DA VIP scores together with Student’s t-test (FC > 1, *p* < 0.05, VIP > 1), and KEGG enrichment was tested by a hypergeometric test.

### 2.5 Statistical analysis

Alpha diversity was estimated in QIIME 2 using observed OTUs, Chao1 richness, and Shannon diversity. Group differences were assessed with the non-parametric Kruskal–Wallis test. Boxplots of *α*-diversity were generated in R (v4.4.1) with ggplot2. Principal coordinate analysis (PCoA) was performed in R using the vegan package. Ordination scatterplots were created with ggplot2, with 95% confidence ellipses to delineate groups.

The phyloseq package was used to calculate the relative abundance of microorganisms at different taxonomic levels (e.g., phylum, genus), and stacked bar charts were plotted to demonstrate the compositional characteristics of the microbial communities by ggplot2. Processing of data and prediction of function by R v4.4.1 tidyverse, microeco, aplot, ggsci, reshape2 packages.

The raw 16S rRNA/ITS sequencing data have been deposited in the NCBI Sequence Read Archive (SRA) under accession number PRJNA1277806. To examine associations between characteristic microbial taxa and key metabolites, Spearman’s rank correlations were computed in R using the psych package. A co-occurrence network was then constructed by retaining only significant associations (|*r*| > 0.7, *p* < 0.05) and visualized in Cytoscape (v3.10.1) (Smoot et al., 2011).

## 3 Results

### 3.1 Analysis of differences in the composition of bacterial microbial communities

#### 3.1.1 Analysis of microbial diversity differences

**Figure 1:**
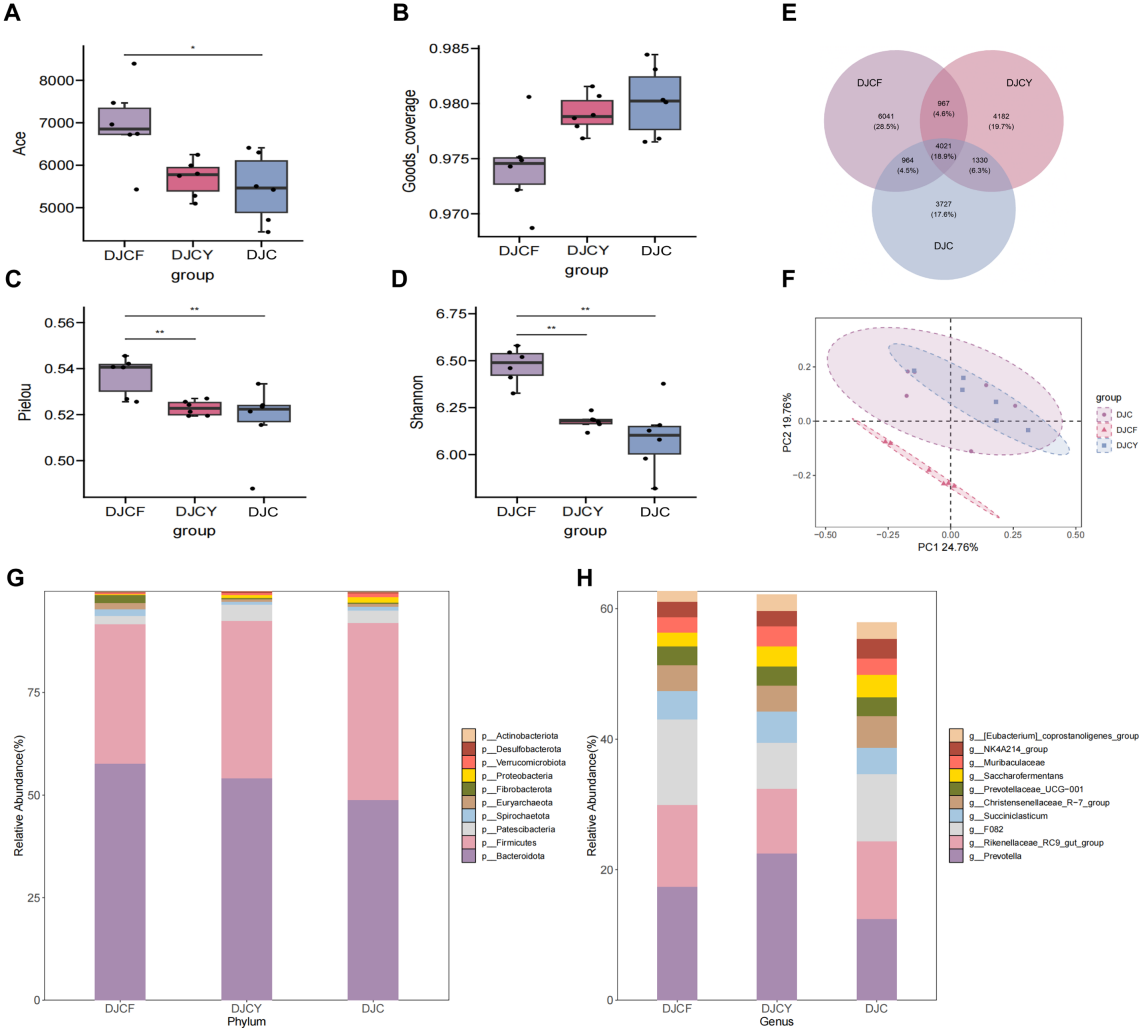
Differential analysis of bacterial microbial communities and diversity. **(A)** Ace index; **(B)** good_coverage index; **(C)** pielou index; **(D)** Shannon index, significant is marked by *p < 0.05, **p < 0.01; **(E)** Venn diagram analysis among groups; **(F)** PCA analysis; The relative abundance of fungal at the phylum level **(G)** and genus level **(H)**.

Dietary composition exerted a pronounced effect on microbial diversity. Alpha-diversity analyses revealed differences among the three groups: the DJCF group showed significantly higher ACE, Pielou’s evenness, and Shannon indices than DJCY and DJC (*p* < 0.05), whereas DJCY and DJC did not differ (*p* > 0.05) (Figures 1A–D). Figure 1E shows that a total of 21,232 OTUs were identified across all dietary treatments: 6,041 in DJCF, 4,182 in DJCY, and 3,727 in DJC. Pairwise overlaps included 967 OTUs shared by DJCF-DJCY, 1,330 by DJCY-DJC, and 964 by DJCF-DJC, with 4,021 OTUs common to all three groups. Principal coordinate analysis (PCoA) further separated DJCF from the other two groups based on bacterial community composition, with PC1 and PC2 explaining 24.76 and 19.76% of the variance, respectively (Figure 1F).

#### 3.1.2 Analysis of bacterial communities in shorthorn cattle

Across dietary treatments, Bacteroidetes and Firmicutes were the dominant phyla, as shown in Figure 1G and Table 2. The relative abundances of Bacteroidetes in the DJCF, DJCY, and DJC groups were 57.62, 54.11, and 48.84%, respectively. Firmicutes was highest in the DJC group (43.08%), followed by DJCY (38.31%) and DJCF (34.07%). However, the differences in the relative abundances of Bacteroidetes and Firmicutes among the three groups were not statistically significant (*p* > 0.05). Among the other phyla, the relative abundance of Patescibacteria was significantly higher in DJCY than in DJCF (*p* < 0.05), whereas no significant difference was detected between DJCY and DJC (*p* > 0.05). By contrast, Fibrobacterota was significantly more abundant in DJCF than in both DJCY and DJC (*p* < 0.05). By contrast, Desulfobacterota was significantly less abundant in DJCF than in DJCY and DJC (*p* < 0.05). Dietary variation influenced the genus-level composition of the microbiota in the DJCF, DJCY, and DJC groups. Across the groups, *Prevotella*, *Rikenellaceae_RC9_gut_group*, and *F082* were the most abundant taxa. *Prevotella* was most abundant in the DJCY group (22.52%) and was significantly more abundant than in the DJC group (*p* < 0.05). *Rikenellaceae_RC9_gut_group* was most abundant in the DJCF group (12.56%), the highest among the three groups (Figure 1H and Table 3).

**Table 2:** Analysis of community composition at the rumen bacterial phylum level of different feeding practices in Shorthorn cattle.

Taxon (phylum level)	DJCF	DJCY	DJC
p__Bacteroidota	57.62 ± 7.45	54.11 ± 10.00	48.84 ± 10.69
p__Firmicutes	34.07 ± 5.25	38.31 ± 9.41	43.08 ± 8.28
p__Patescibacteria	1.98 ± 0.64^b^	4.04 ± 1.46^a^	3.08 ± 0.95^ab^
p__Spirochaetota	1.66 ± 1.52	0.63 ± 0.32	0.93 ± 0.33
p__Euryarchaeota	1.44 ± 1.05	0.68 ± 0.37	0.70 ± 0.50
p__Fibrobacterota	2.02 ± 1.82^a^	0.24 ± 0.07^b^	0.32 ± 0.16^b^
p__Proteobacteria	2.16 ± 0.62	3.03 ± 1.31	3.47 ± 2.03
p__Verrucomicrobiota	0.17 ± 0.08	0.78 ± 0.79	1.31 ± 1.55
p__Desulfobacterota	0.29 ± 0.13^b^	0.53 ± 0.52^a^	0.92 ± 1.19^a^
p__Actinobacteriota	0.18 ± 0.09	0.08 ± 0.04	0.15 ± 0.12

Means within a row with different superscripts differ significantly at *p* < 0.05.

**Table 3:** Analysis of community composition at the rumen bacterial genus level of different feeding practices in Shorthorn cattle.

Taxon (genus level)	DJCF	DJCY	DJC
g__Prevotella	17.39 ± 5.52^ab^	22.49 ± 9.79^a^	12.47 ± 3.86^b^
g__Rikenellaceae_RC9_gut_group	12.55 ± 2.06	9.93 ± 3.78	11.89 ± 5.96
g__F082	13.09 ± 2.34	7.04 ± 2.18	10.34 ± 8.85
g__Succiniclasticum	4.43 ± 2.57	4.74 ± 2.94	4.07 ± 3.40
g__Christensenellaceae_R-7_group	3.90 ± 0.49	3.93 ± 2.26	4.76 ± 1.41
g__Prevotellaceae_UCG-001	2.88 ± 0.62	3.01 ± 0.94	2.90 ± 0.76
g__Saccharofermentans	2.16 ± 0.62	3.03 ± 1.31	3.47 ± 2.03
g__Muribaculaceae	2.37 ± 1.87	3.13 ± 2.92	2.52 ± 1.69
g__NK4A214_group	2.30 ± 0.50	2.36 ± 0.91	2.96 ± 0.55
g__[Eubacterium]_coprostanoligenes_group	1.67 ± 0.51^b^	2.53 ± 0.63^a^	2.52 ± 0.75^a^

Means within a row with different superscripts differ significantly at *p* < 0.05.

#### 3.1.3 Microbial interactions between rumen bacterial communities in shorthorn cattle

**Figure 2:**
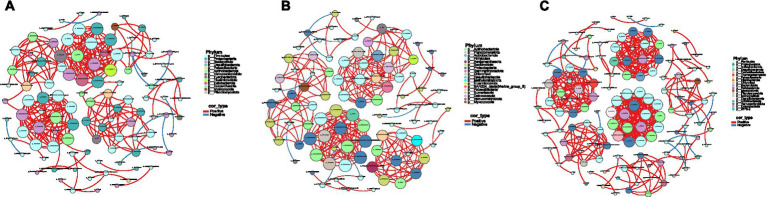
Network diagram. Different nodes represent different genera, node size represents the average relative abundance of the genus, nodes of the same phylum have the same color (as indicated in the figure legend), the absolute value of the correlation coefficient between line thickness and species interactions between nodes is positive, and the correlation coefficient between line color and correlation is positive (red is positively, blue is negatively). **(A)** DJCF, **(B)** DJCY, **(C)** DJC.

Correlation analysis (Figures 2A–C) depicts co-occurrence networks of the rumen microbiota in Shorthorn cattle under different diets (DJCF, DJCY, DJC). Network analysis revealed significantly more positive than negative correlations among congeneric taxa (*p* < 0.05). Notably, taxa within the same genus showed strong positive associations (*p* < 0.05).

### 3.2 Analysis of differences in the composition of fungal microbial communities

#### 3.2.1 Analysis of fungal diversity differences

**Figure 3:**
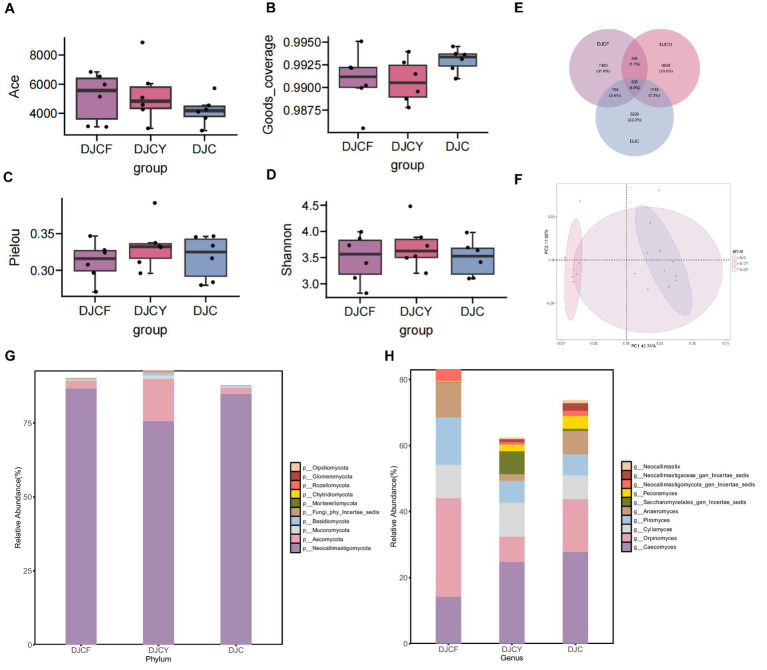
Differential analysis of fungal microbial communities and diversity. **(A)** Ace index; **(B)** good_coverage index; **(C)** pielou index; **(D)** Shannon index, significant is marked by*p < 0.05, **p < 0.01; **(E)** Venn diagram analysis among groups; **(F)** PCA analysis; The relative abundance of fungal at the phylum level **(G)** and genus level **(H)**.

*α*-Diversity analysis (Figures 3A–D) revealed no significant differences in α-diversity indices among the three groups (*p* > 0.05). Venn-diagram analysis of shared and unique features (Figure 3E) identified 23,478 features across all groups, of which 939 were shared. The DJCF, DJCY, and DJC groups contained 7,483, 6,939, and 5,208 unique OTUs, respectively.

*β*-Diversity analysis using PCoA (Figure 3F) showed that the fungal community composition of the DJCF group was distinct from the other two groups, while DJCY and DJC did not differ. The first two principal coordinates (PC1 and PC2) accounted for 46.74 and 11.02% of the total variation, respectively. Samples from the DJCF and DJCY groups formed tight clusters, indicating lower within-group variability. Conversely, samples from the DJC group were more dispersed, suggesting higher within-group variability.

#### 3.2.2 Analysis of fungal microbial communities

A comparison of the dominant phyla among the three groups is shown in Figure 3G and Table 4. We analyzed the 10 identified fungal phyla. In the DJCF, DJCY, and DJC groups, the dominant phyla were Neocallimastigomycota and Ascomycota. Neocallimastigomycota accounted for 86.74, 75.68, and 84.88% in DJCF, DJCY, and DJC, respectively; Ascomycota accounted for 2.54, 14.25, and 2.09%, respectively. No significant differences in the relative abundances of these dominant phyla were detected among the three groups (*p* > 0.05). As shown in Figure 3H and Table 5, *Caecomyces* accounted for 14.22, 24.73, and 27.85% in DJCF, DJCY, and DJC, respectively, and was highest in DJC; its abundance in DJC was significantly higher than in DJCF (*p* < 0.05). *Orpinomyces* was most abundant in DJCF (29.83%) and was significantly higher than in DJCY (7.63%) and DJC (15.98%) (*p* < 0.05). *Cyllamyces* accounted for 10.13, 10.24, and 7.24% in DJCF, DJCY, and DJC, respectively, and *Piromyces* accounted for 14.26, 6.62, and 6.30%, respectively, there were no significant differences among the three groups (*p* > 0.05).

**Table 4:** Rumen fungi community composition at phylum in the different feeding systems in Shorthorn cattle.

Taxon (phylum level)	DJCF	DJCY	DJC
p__Neocallimastigomycota	86.74 ± 15.04	75.68 ± 21.48	84.88 ± 20.31
p__Ascomycota	2.54 ± 2.66	14.25 ± 16.41	2.09 ± 2.14
p__Mucoromycota	0.35 ± 0.43^b^	1.15 ± 0.87^a^	0.34 ± 0.30^b^
p__Basidiomycota	0.31 ± 0.26	1.05 ± 1.05	0.39 ± 0.43
p__Fungi_phy_Incertae_sedis	0.18 ± 0.26^ab^	0.57 ± 0.56^a^	0.09 ± 0.07^b^
p__Mortierellomycota	0.002 ± 0.01	0.03 ± 0.05	0.002 ± 0.002
p__Chytridiomycota	0.004 ± 0.005	0.01 ± 0.01	0.003 ± 0.004
p__Rozellomycota	0.002 ± 0.003	0.003 ± 0.005	0.002 ± 0.003
p__Glomeromycota	0.00 ± 0.00	0.005 ± 0.01	0.00 ± 0.00
p__Olpidiomycota	0.001 ± 0.002	0.00 ± 0.00	0.00 ± 0.00

Means within a row with different superscripts differ significantly at *p* < 0.05.

**Table 5:** Rumen fungi community composition at genus in the different feeding systems in Shorthorn cattle.

Taxon (genus level)	DJCF	DJCY	DJC
g__Caecomyces	14.23 ± 5.44^b^	24.75 ± 11.55^ab^	27.79 ± 9.34^a^
g__Orpinomyces	29.86 ± 8.95^a^	7.70 ± 4.97^b^	15.93 ± 15.41^b^
g__Cyllamyces	10.11 ± 5.78	10.22 ± 8.92	7.18 ± 5.68
g__Piromyces	14.34 ± 14.63	6.65 ± 3.89	6.37 ± 8.66
g__Anaeromyces	10.62 ± 4.02^a^	1.98 ± 1.30^b^	7.16 ± 7.24^ab^
g__Saccharomycetales_gen_Incertae_sedis	0.14 ± 0.22	7.05 ± 12.25	0.65 ± 1.03
g__Pecoramyces	0.30 ± 0.28^b^	1.91 ± 1.06^ab^	3.85 ± 3.87^a^
g__Neocallimastigomycota_gen_Incertae_sedis	3.38 ± 2.07^a^	0.71 ± 1.07^b^	1.58 ± 1.37^ab^
g__Neocallimastigaceae_gen_Incertae_sedis	0.00 ± 0.00^b^	0.94 ± 0.61^b^	2.27 ± 1.77^a^
g__Neocallimastix	0.00 ± 0.00^b^	0.45 ± 0.43^ab^	0.95 ± 0.81^a^

Means within a row with different superscripts differ significantly at *p* < 0.05.

#### 3.2.3 Microbial interactions between rumen fungal communities in Shorthorn cattle

**Figure 4:**
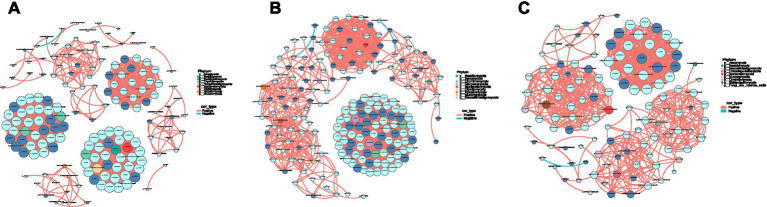
Network diagram. Different nodes represent different genera, node size represents the average relative abundance of the genus, nodes of the same phylum have the same color (as indicated in the figure legend), the absolute value of the correlation coefficient between line thickness and species interactions between nodes is positive, and the correlation coefficient between line color and correlation is positive (red is positively, blue is negatively). **(A)** DJCF, **(B)** DJCY, **(C)** DJC.

To elucidate the relationships among the DJCF, DJCY, and DJC microbial communities in Shorthorn cattle under different diets, we constructed co-occurrence networks (Figures 4A–C). Network analysis showed that positive correlations between species were more frequent than negative ones, particularly among congeneric taxa.

### 3.3 Rumen metabolome associated with different feeding patterns

#### 3.3.1 Metabolomic profiles in rumen

**Figure 5:**
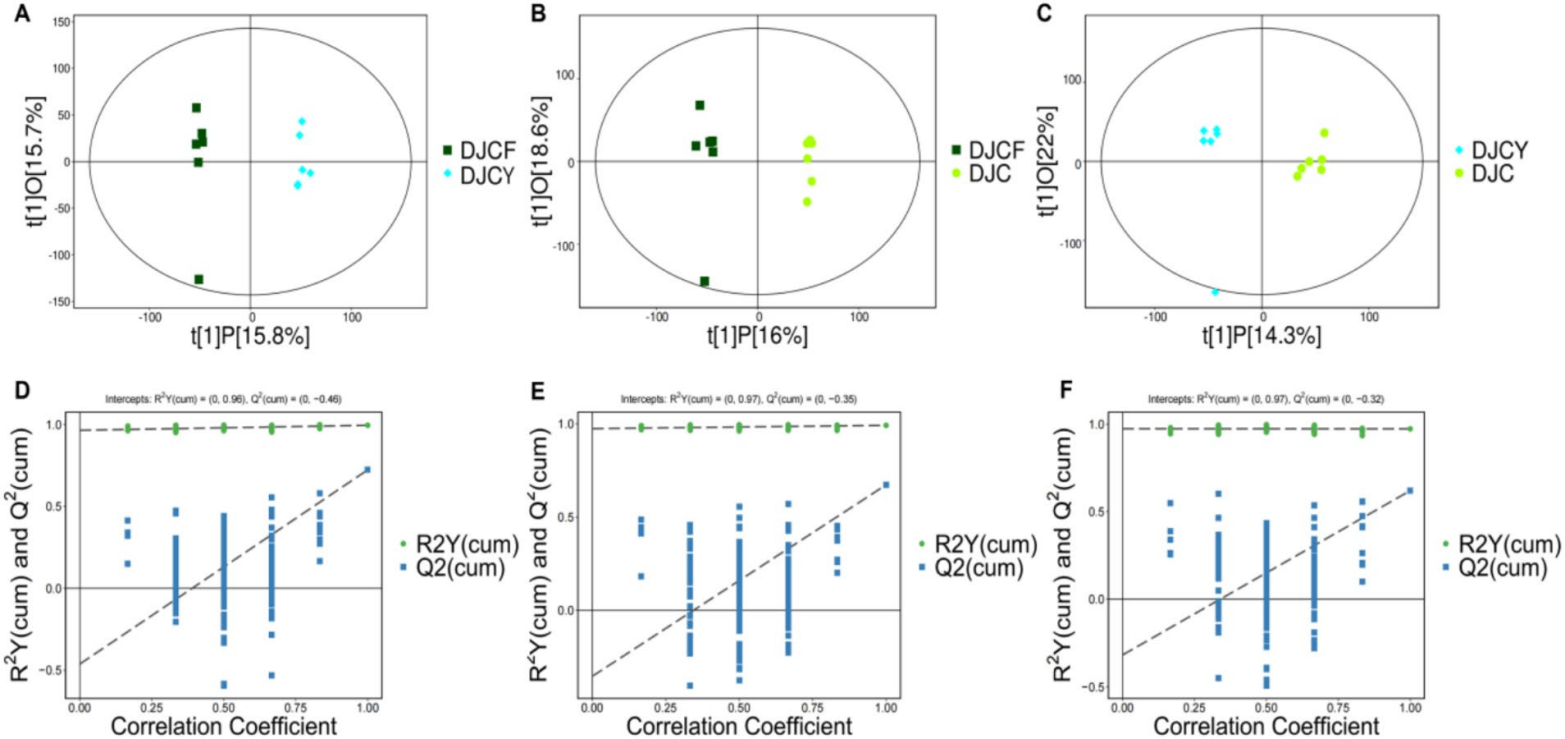
Corresponding validation plots and orthogonal projections to latent structures-discriminant analysis (OPLS-DA) score plots derived from gas chromatography time-of-flight mass spectrometer (GC-TOF/MS) metabolite profiles of Shorthorn cattle rumen samples with Different feeding methods. Corresponding validation plots and OPLS-DA score plots for **(A,D)** group DJCF vs. group DJCY, **(B,E)** group DJCF vs. group DJC, and **(C,F)** group DJCY vs. DJC.

We profiled rumen metabolites using untargeted metabolomics. Orthogonal partial least squares–discriminant analysis (OPLS-DA) showed marked differences among the DJCF, DJCY, and DJC groups (Figures 5A–C). Pairwise comparisons were well separated in the score plots, and all samples fell within the 95% confidence ellipse. As shown in Figures 5D–F, the R^2^Y/Q^2^ values for the comparisons between the DJCF and DJCY group, the DJCF and DJC group, and the DJCY and DJC group were 0.995/0.723, 0.992/0.673, and 0.973/0.620, respectively. These results indicate good model fit (R^2^Y) and predictive ability (Q^2^), supporting the reliability of subsequent statistical analyses.

#### 3.3.2 Analysis of differential metabolites under different feeding practices

**Figure 6:**
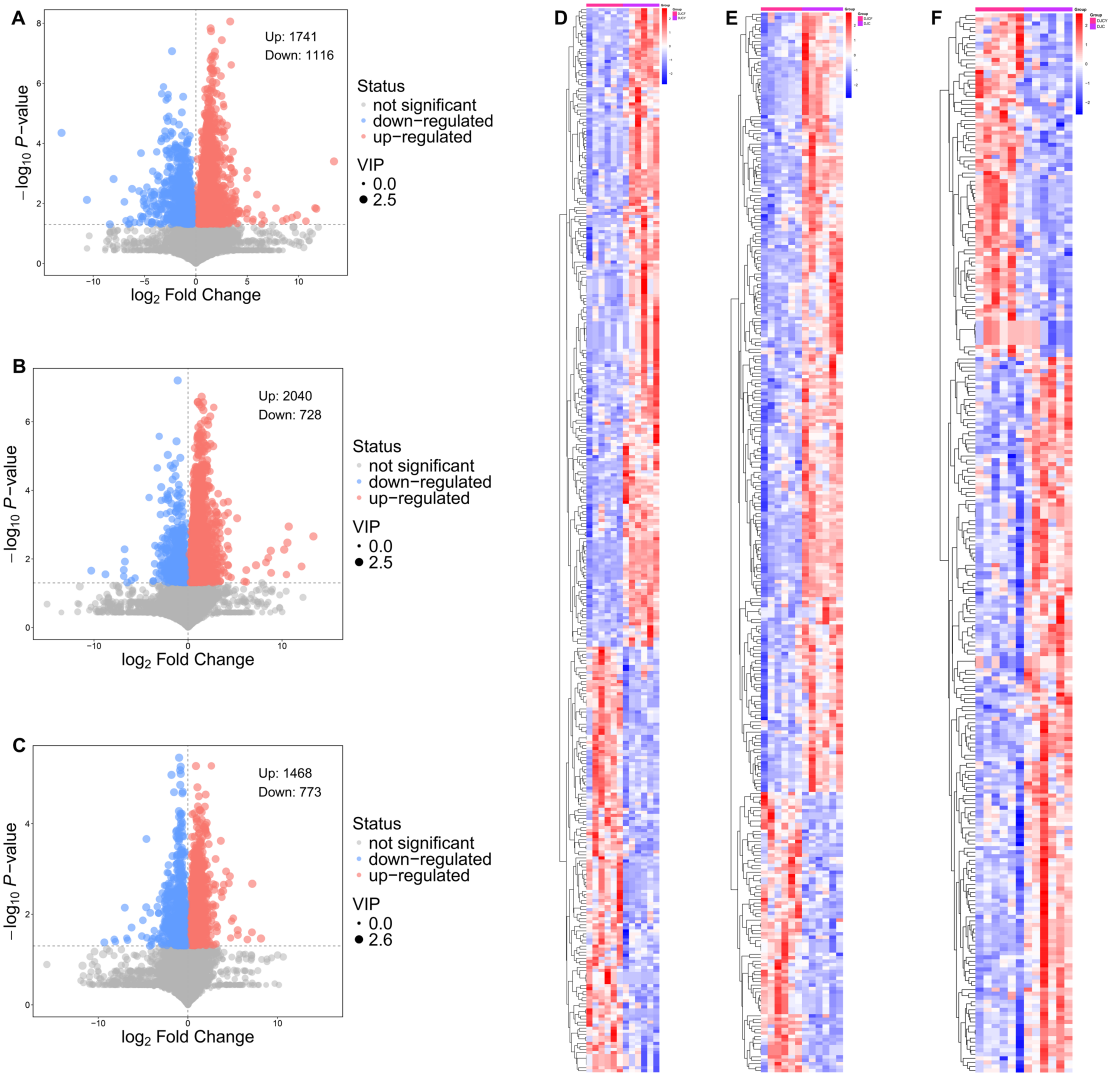
Ruminal differential metabolites identified by volcano plots **(A–C)** and hierarchical clustering analysis **(E,F)** in Shorthorn cattle under different feeding practices. Comparisons: **(A,D)** DJCF vs. DJCY, **(B,E)** DJCF vs. DJC, **(C,F)** DJCY vs. DJC.

As shown in Figures 6A–C, 116 metabolites were downregulated and 1741 metabolites were upregulated in DJCF vs. DJCY group. Seven hundred and twenty-eight metabolites were downregulated and 2040 metabolites were upregulated in DJCF vs. DJC group. Seven hundred and seventy-three metabolites were downregulated and 1,468 metabolites were upregulated in DJCY vs. DJC group. DJCY group contained 45 metabolites with significantly elevated concentrations compared to DJCF group (*p* < 0.05, VIP > 1), such as Daltogen, Cytidine 5′-monophosphate (CMP), 1-Methyladenosine, Turanose, Isomaltose, Lactose, Cellobiose, Zidovudine. There were 28 metabolites with significantly different properties in the DJCF group that were significantly higher than in the DJCY group (*p* < 0.05), such as 2-methylbenzoic acid, 4-methylbenzoic acid, phenylacetic acid, trimethylamine N-oxide, and caprolactam. The comparison analysis in the DJCF vs. DJC group revealed that the relative concentrations of 47 metabolites [e.g., Octanoylglucuronide, 2’-Deoxyguanosine 5′-monophosphate (dGMP), Adenosine monophosphate (AMP), 2’-Deoxyadenosine 5′-monophosphate (dAMP) and Adenosine] were significantly higher in the rumen of DJC group, and the relative concentrations of 18 metabolites [e.g., 2-Oxoadipic acid, Luteolin, Prostaglandin A2, and Prostaglandin B2 (PGB2) were significantly higher in the DJCF group] (*p* < 0.05, VIP > 1). A total of 27 metabolites, such as Octanoylglucuronide, Eupatilin, Astragalin, 5-Hydroxyvalproic acid and Kynurenic acid were abundant in the DJC group, and 2-Hydroxybutyric acid, Prostaglandin A2, Prostaglandin B2 (PGB2), 13(S)-HODE, and PC (16:0/P-16:0) were abundant in the DJCY group by the comparison analysis in the DJCY vs. DJC group (Figures 6D–F).

#### 3.3.3 Metabolic pathways of differential metabolites

**Figure 7:**
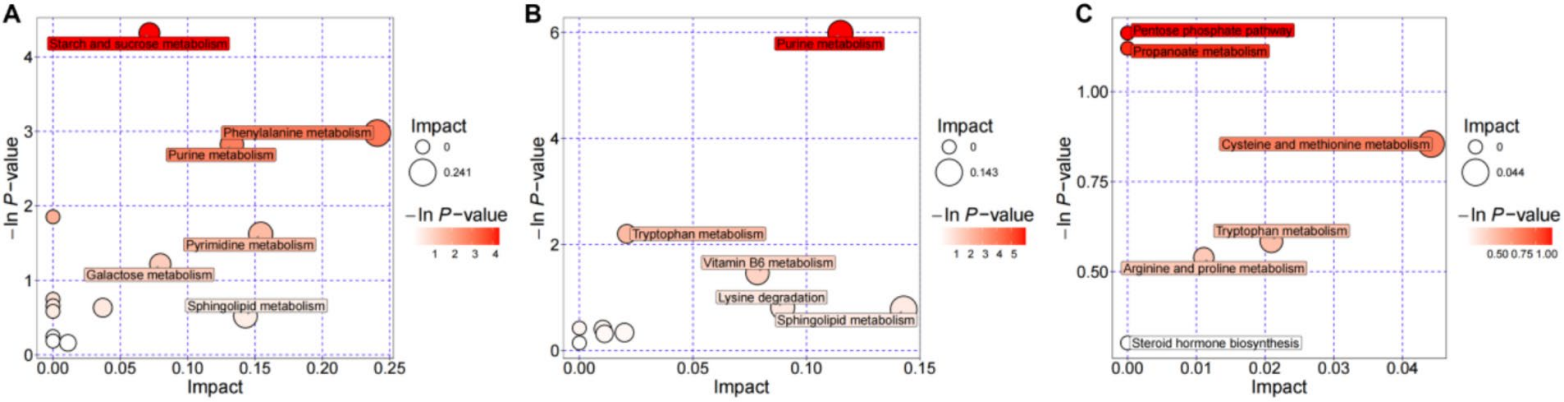
Pathway enrichment analysis using significant differences in rumen metabolites of Shorthorn cattle under different feeding practices. **(A)** DJCF vs. DJCY, **(B)** DJCF vs. DJC, **(C)** DJCY vs. DJC.

Using MetPA, we identified 14 metabolic pathways in the DJCF vs. DJCY comparison; among these, the starch and sucrose metabolism pathway and the purine metabolism pathway were significant. In the DJCY vs. DJC comparison, six pathways were identified, but none reached significance (*p* < 0.01, pathway impact > 0.1) (Figures 7A–C). According to KEGG mapping, cellobiose, trehalose, maltose, and isomaltose mapped to the starch and sucrose metabolism pathway, whereas adenosine 5′-diphosphate (ADP), adenosine monophosphate (AMP), adenosine, 2′-deoxyadenosine 5′-monophosphate (dAMP), deoxyinosine, deoxyguanosine, and adenine mapped to purine metabolism.

#### 3.3.4 Correlations between the ruminal metabolomes and microbiomes

**Figure 8:**
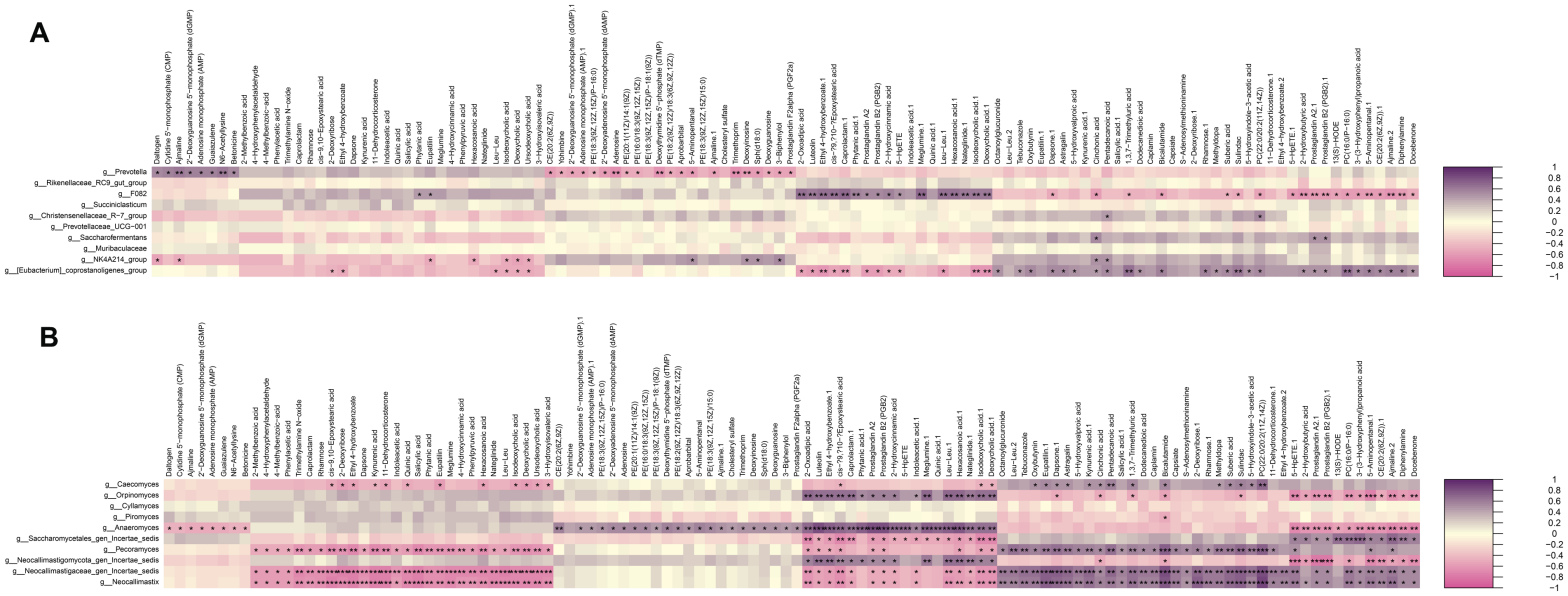
Correlation analysis between genera and metabolite concentrations affected by the different feeding methods. **(A)** Correlation between differential bacteria and differential metabolites. **(B)** Correlation between differential fungi and differential metabolites.

To investigate links between the rumen microbiota and host metabolism, we assessed correlations between the 10 most abundant bacterial genera and the 10 most abundant fungal genera and metabolites detected in rumen fluid using Spearman’s rank correlation analysis (Figures 8A,B). Among bacterial genera, *Prevotella* showed significantly positive correlations with eight metabolites, including daltogen, cytidine 5′-monophosphate (CMP), N6-acetyllysine, betonicine, and 2′-deoxyguanosine 5′-monophosphate (dGMP), and significantly negative correlations with 20 metabolites, including yohimbine, PC (14:0/P-16:0), sebacic acid, 5-hydroxyindole-3-acetic acid, trimethoprim, Zeran, and adenine (*p* < 0.05). *gF082* showed significant positive correlations with phytanic acid, eupatilin, 2-oxoadipic acid, luteolin, ethyl 4-hydroxybenzoate, and caprolactam (*p* < 0.05)*. g_NK4A214_group* showed significant positive correlations with six differentially abundant metabolites, and *g_[Eubacterium]_coprostanoligenes_group* with 27 differentially abundant metabolites (*p* < 0.05) (Figure 8A).

Among fungal genera (Figure 8B), *Caecomyces, Neocallimastigaceae gen. Incertae sedis*, and *Neocallimastix* showed positive correlations with 14 metabolites, including eupatilin, PC (22:0/20:2(11Z,14Z)), PC (16:0/P-16:0), astragalin, suberic acid, 5-aminopentanoic acid, S-adenosylmethioninamine, and ethyl 4-hydroxybenzoate. Conversely, *Orpinomyces* was significantly negatively associated with these differentially abundant metabolites (*p* < 0.05). *Saccharomycetales_gen_Incertae_sedis* was positively associated with 24 metabolites (*p* < 0.05), such as cytidine 5′-monophosphate (CMP), zidovudine, 8-hydroxycarbostyril, trehalose, PC (16:0/16:0), diphenylamine, and turanose.

## 4 Discussion

The microbial community within the rumen is essential for sustaining the physiological health and normal digestive function of ruminants (Ley et al., 2008). In this study, our multi-omics analysis revealed distinct shifts in rumen microbiota and metabolomes in Shorthorn cattle under different feeding regimens, thereby providing novel insights into the interplay between dietary factors, microbial dynamics, and metabolic outcomes. The taxonomically rich rumen bacterial community drives anaerobic digestion to generate bioavailable substrates for host metabolism (Saleem et al., 2012; Simon et al., 2017). Alpha diversity serves as a key indicator of microbial community robustness, with higher values indicating greater structural stability and functional resilience against environmental perturbations (Jiang F. et al., 2021). Our results showed that Chao1 and Shannon indices were significantly higher in the naturally grazed group (DJCF) than in the intensively fed groups (DJCY, DJC), consistent with previous findings that high-fiber diets enhance community stability. Liu et al. observed that increasing dietary concentrates reduced rumen microbial diversity in yaks (Liu et al., 2019); similarly, He et al. reported higher microbial diversity in naturally grazed cattle (He et al., 2024a). This effect may stem from a decrease in rumen pH due to high-concentrate diets, which in turn inhibit the growth of fiber-degrading bacteria (Sonnenburg and Bäckhed, 2016). The PCoA results further confirmed the distinct bacterial and fungal community structures between DJCF and the intensive-feeding groups, with the first two principal components explaining 24.76 and 19.76% of bacterial variance, respectively, which indicates a strong dietary impact on microbial assembly.

At the phylum level, we found that Bacteroidota and Firmicutes were dominant; together, these phyla have been reported to comprise more than 80% of the bacteria in the gastrointestinal tract (Eckburg et al., 2005; Jiang C. X. et al., 2021; Liu et al., 2021). These observations are consistent with previous research (Chen et al., 2015; Ma et al., 2023; Zhou et al., 2017). Bacteroidota utilize carbohydrate-active enzymes (CAZymes) to ferment dietary polysaccharides into short-chain fatty acids (SCFAs) while mediating protein, bile acid, and oligosaccharide metabolism (He et al., 2024b; Hess et al., 2011; Thomas et al., 2011). Synergistically, Firmicutes employ specialized hydrolases to bioconvert complex substrates (e.g., cellulose, proteins) into bioavailable nutrients (He et al., 2024b; Huo et al., 2014). This cross-phylum partnership optimizes host digestive efficiency through complementary metabolic pathways (He et al., 2024a; Nagaraja and Titgemeyer, 2007). The DJCF group had the highest Bacteroidota abundance (57.62%) and the lowest Firmicutes abundance (34.07%), while the DJC group showed the opposite trend (Bacteroidota 48.84% and Firmicutes 43.08%). This shift coincided with changes in metabolic profiles: higher Bacteroidota in DJCF was associated with elevated fibre-degradation metabolites (e.g., cellobiose), whereas increased Firmicutes in the intensive-feeding groups correlated with higher purine metabolites (AMP, dAMP) and carbohydrate intermediates (maltose, glucose). These findings suggest that the Bacteroidota–Firmicutes balance acts as a key regulator of nutrient-utilization pathways in response to dietary change.

At the genus level, Prevotella and Rikenellaceae_RC9_gut_group were the dominant taxa, both belonging to Bacteroidetes, in agreement with the results of the present study (He et al., 2024a; Yang et al., 2024). Functionally, Prevotella is a key rumen bacterium mediating proteolysis and amylolysis via secreted enzymes (proteases, amylases, hemicellulases), thereby enabling the degradation of dietary proteins, starch, and hemicellulose; its abundance typically increases with higher dietary protein (Bekele et al., 2010; Cholewińska et al., 2020; He et al., 2024a). *Rikenellaceae_RC9_gut_group*, in turn, has been linked to lipid metabolism and the production of short-chain fatty acids, notably propionate, and may also contribute to protein degradation (Conte et al., 2022; Fan et al., 2017). Diet appears to modulate the relative dominance of these genera: Fernando et al. reported that concentrate-fed beef cows had a higher proportion of Bacteroidetes than those fed green hay, with *Rikenellaceae_RC9_gut_group* as the dominant genus (Fernando et al., 2010; He et al., 2024a), whereas in our study *Prevotella* predominated in the DJCY and DJC groups. This discrepancy likely reflects the higher crude-protein and starch contents of the DJCY/DJC total mixed-ration diets, which favor protein- and starch-degrading microbes such as *Prevotella*. Correlation analyses further supported these functional inferences: *Prevotella*, most abundant in DJCY (22.52%), showed significant positive correlations with cytidine 5′-monophosphate (CMP) and 2′-deoxyguanosine 5′-monophosphate (dGMP), and negative correlations with phytanic acid and caprolactam, suggesting a context-specific role in nucleotide metabolism under intensive feeding. By contrast, *Rikenellaceae_RC9_gut_group*, most abundant in DJCF (12.56%), associated with higher levels of propionate precursors, consistent with its contribution to lipid metabolism and short-chain fatty acid (SCFA) synthesis (Conte et al., 2022). Taken together, these patterns align with evidence that altering feed type and concentrate proportion (e.g., TMR with higher crude protein and starch) reshapes the Bacteroidetes/Firmicutes balance and modulates carbohydrate and purine-related pathways, thereby favoring *Prevotella*-driven metabolism under intensive diets.

Anaerobic fungi are pivotal decomposers in the rumen, and *Neocallimastigomycota* typically predominate; in our dataset they accounted for 75.68–86.74% of the fungal community, in line with reports that this phylum is the dominant fungal group in ruminants (Boots et al., 2013; Denman et al., 2018; Wallace et al., 2019). Diet remodels fungal diversity and composition: intensive feeding reshapes the rumen microbiota, including the mycobiome, whereas seasonal and forage quality shifts also modulate fungal taxa (Cengiz and John, 2002; Faichney et al., 1997; He et al., 2024b; Wang et al., 2023). In our study, the natural-grazing group (DJCF) displayed higher Neocallimastigomycota abundance than the intensive-feeding groups, consistent with literature showing context-dependent fungal responses to dietary inputs in high-altitude ruminants. Functionally, *Neocallimastigomycota* enhance nutrient bioavailability by secreting potent cellulases and cellulosomes that depolymerize recalcitrant plant cell walls (e.g., Orpinomyces systems) (Abhaya et al., 2017; Boots et al., 2013). Beyond classic fiber degradation, our integrative analyses revealed fungus–metabolite couplings that highlight ecological specialization: *Orpinomyces* (enriched in DJCF) correlated positively with 2-oxoadipic acid and luteolin—metabolites linked to plant-matrix breakdown—whereas *Caecomyces* (elevated under intensive feeding) correlated with phosphatidylcholine (PC) species, suggesting adjustment of lipid-related metabolism under high-concentrate diets. These findings extend current evidence that feed type and concentrate proportion restructure the rumen community and its metabolic outputs, and they pinpoint fungal lineages that track specific metabolite pathways, thereby underscoring fungi’s contribution to host energy harvest beyond lignocellulose turnover (Denman et al., 2018; He et al., 2024a; He et al., 2024b; Wallace et al., 2019).

Intensive or high-concentrate feeding reshapes rumen carbohydrate metabolism and fermentation; in yaks, multi-omics shows that intensive systems elevate dietary starch/protein and reconfigure the microbiome–metabolome interface (Yang et al., 2024). Consistent with this paradigm, our intensive-feeding groups (DJCY/DJC) accumulated carbohydrate intermediates (e.g., maltose, glucose), and pathway analysis indicated significant shifts in starch and sucrose metabolism together with purine metabolism, a pattern widely observed when feed type or forage-to-concentrate ratios change (Liu et al., 2019; Penner et al., 2009). Similar to the results of this experiment, we observed a significant increase of carbohydrate metabolites in the DJCY group, such as maltose, glucose, which are important intermediates of carbohydrate metabolism. The metabolic pathway is primarily sucrose and starch metabolism. In the rumen, microbial enzymes break down starch and cellulose into glucose, which then undergoes glycolysis to produce pyruvate. This pyruvate, derived from carbohydrate metabolites, acts as the key substrate for VFA production (Xue et al., 2018). Metabolic pathway analysis revealed that sucrose and starch metabolism and purine metabolism were significantly affected by feeding regimens. Four metabolites (Cellobiose, Trehalose, Maltose, Isomaltose) in the sucrose and starch pathway were more abundant in DJCF, aligning with the higher fiber-degrading microbiota in this group. Meanwhile, purine metabolites (AMP, dAMP, Adenosine) were enriched in DJCY and DJC, which corresponded to the elevated *Prevotella* abundance and were consistent with the role of these metabolites as biomarkers for microbial protein synthesis (Ametaj et al., 2010). The significant correlation between *Prevotella* and purine metabolites further confirms the direct involvement of specific taxa in regulating these pathways. Notably, our study identified several low-abundance microbial taxa with potential functional significance. LEfSe analysis revealed nine differential species in DJCF, including *Fibrobacter succinogenes*, which showed positive correlations with phytanic acid and eupatilin despite its low relative abundance. Similarly, the DJC group had specific low-abundance oscillospiraceae taxa associated with octanoylglucuronide, a metabolite involved in lipid conjugation. These findings highlight that even low-abundance microbes may play critical roles in metabolic regulation, but their actual metabolic functions remain unclear due to limitations in current multi-omics approaches.

Of particular interest is the discovery of changes in several metabolites associated with the purine metabolic pathway. Ruminal microbes degrade nitrogenous compounds from feed and channel them into nucleic-acid metabolism through *de novo* and salvage pathways; nucleosides are then catabolized to bases (e.g., xanthine, hypoxanthine, guanine, adenine) and further to degradation products (Fujihara and Shem, 2011; Stentoft et al., 2015). We found higher levels of purine-related metabolites in the concentrate-fed groups, consistent with previous reports of elevated xanthine and hypoxanthine under high-concentrate diets (Ametaj et al., 2010; Zhang et al., 2017). In addition, adenine and xanthine—and likely pseudouridine—identified among the feed-intake groups likely originate from bacterial nucleic-acid degradation (Noel et al., 2017; Sutton et al., 1975). In the above results, it was shown that concentrate feed contributes to rumen purine metabolism. Arachidonic acid functions as a sensitive and specific plasma biomarker for the average daily gain (ADG) in steers (Artegoitia et al., 2016). Furthermore, Naturally occurring in all mammalian cells, arachidonic acid (an *ω*-6 tetra-unsaturated fatty acid) maintains membrane fluidity while serving as the primary biosynthetic precursor for lipid-derived signaling molecules, including prostaglandins and leukotrienes (Martin et al., 2016). In this study, we found a higher concentration of prostaglandin in the DJCF, DJCY group compared with that in the DJC group. It may be related to the effect of castration of Shorthorn cattle on androgen. Phosphatidylcholine (PC), a glycerophospholipid, serves as a critical structural element in cellular lipid bilayers. Choline constitutes a primary component of PC, and adequate PC levels are essential for supporting lipid transport mechanisms (Shiau and Cho, 2002; Yeh et al., 2015). PC functions as a choline reservoir, with the stored choline being further processed into organic osmolytes through metabolic pathways involving glycine, serine, and threonine (Athamena et al., 2011; Jiang et al., 2018). Ren et al. showed that PC concentration increased linearly with increasing concentrate levels and that this metabolite, which is related to lipid metabolism, plays an important role in yak growth and rumen health. The results of the present experimental study showed that the PC concentration in the two fattening groups were significantly higher than that in the grazing group, indicating that the PC concentration increased with the nutrient level of the diets. To further explore the potential connection between rumen dominant microflora, and metabolites, Pearson correlation analysis was carried out. Dai et al. indicated that differential metabolites are significantly correlated, either positively or negatively, with dominant microorganisms (Dai et al., 2023). Emerging evidence from integrated multi-omics investigations has revealed utilization or production associations between bacteria and metabolomes in the rumen (Liu et al., 2019; Ma et al., 2021). *Saccharomycetales_gen_Incertae_sedis* in the DJCY group showed positive correlations with 24 metabolites (including CMP and turanose), implying a potential role in nucleotide and carbohydrate metabolism. However, the regulatory mechanisms driving these associations—whether through direct metabolic conversion or indirect ecological interactions—remain to be elucidated.

Overall, these patterns highlight the effects of different feeding strategies in Shorthorn cattle and provide insights into the mechanisms by which feeding practices shape rumen microbial processes. While our study integrates 16S rRNA gene and ITS amplicon sequencing with metabolomics, directly inferring *in situ* metabolic functions and the specific regulatory roles of low-abundance bacteria and fungi remains challenging. In future work, we will combine metagenomic and metatranscriptomic approaches to delineate links between microbial gene expression and metabolic pathways, and we will elucidate organism-level functions in the rumen microecosystem through isolation and in-vitro metabolic validation of key strains, thereby providing a theoretical basis for precision nutrition in Shorthorn cattle.

## 5 Conclusion

This study used multi-omics analysis to reveal the adaptive mechanisms of the rumen microbiome and metabolites of Shorthorn cattle in intensive feeding systems. High-concentrate diets enriched proteolytic bacteria *Prevotella* and suppressed fiber-degrading fungi *Neocallimastigomycota*, activating purine metabolism pathways, indicating enhanced microbial protein synthesis efficiency. Concurrently, Shorthorn cattle maintained Bacteroidota functionality under high-starch diets through host genetic adaptation. Thus, an intensive feeding system altered the rumen microbiome and rumen metabolism, resulting in improvements of Shorthorn cattle growth. These findings provide novel strategies for precision feeding based on metabolic biomarkers, though further validation is required to address limitations such as limited sample size and potential confounding effects of castration.

## Data Availability

The datasets presented in this study can be found in online repositories. This data can be found here: https://www.ncbi.nlm.nih.gov/, PRJNA1277806.

## Footnotes

^1^ http://ccb.jhu.edu/software/FLASH/
